# A Case of Anti-3-Hydroxy-Methylglutaryl-Coenzyme a Reductase (Anti-HMGCR) Immune-Mediated Necrotizing Myopathy

**DOI:** 10.7759/cureus.97703

**Published:** 2025-11-24

**Authors:** Jennifer S George, Sahil Sabharwal, Robert Donnell, Benjamin M Boral

**Affiliations:** 1 Internal Medicine, University of Arkansas for Medical Sciences, Fayetteville, USA; 2 Internal Medicine, University of Arkansas for Medical Sciences/Mercy Hospital, Arkansas, USA

**Keywords:** anti-hmgcr antibodies, immune-mediated necrotizing myopathy, myositis, proximal muscle involvement, statin

## Abstract

Anti-3-hydroxy-methylglutaryl-coenzyme A reductase (Anti-HMGCR) immune-mediated necrotizing myopathy (INMN) is a type of autoimmune myopathy that is commonly associated with a history of statin use. In this case report, we present a 47-year-old man with a history of statin use who was admitted to the hospital with elevated creatine kinase and progressive muscle weakness. Muscle biopsy and myositis panel confirmed the diagnosis of anti-HMGCR IMNM. Creatine kinase levels and symptoms improved with the initiation of glucocorticoids, mycophenolate mofetil, and rituximab. This case report expresses the need for continued research into the treatment guidelines as well as the long-term effects of anti-HMGCR IMNM.

## Introduction

Immune-mediated necrotizing myopathy (IMNM) is a subtype of idiopathic inflammatory myopathy that is characterized by proximal muscle weakness, elevated levels of creatine kinase, and necrosis in the absence of a significant inflammatory cell infiltrate. These diseases are further categorized based on the antibodies found in the blood, particularly anti-signal recognition particle (SRP), anti-3-hydroxy-methylglutaryl-coenzyme A reductase (HMGCR), or seronegative IMNM [1].

Compared to the other subgroups of non-IMNM inflammatory myopathies, anti-HMGCR IMNM has one of the lowest prevalences, which is estimated to range from 6% to 12% of all the IMNMs [2], although this statistic may be underestimated due to a lack of awareness of the disease and access to testing to confirm the disease. Despite the fact that studies have shown that nearly 30% of people with anti-HMGCR IMNM have never taken a statin, the increasing prevalence of statins highlights the importance of physicians to recognize the disease in order to provide prompt treatment [3,4]. This case report focuses on one of these presentations of statin-associated anti-HMGCR-positive IMNM.

## Case presentation

A 47-year-old male with a past medical history of type 2 diabetes mellitus, hypertension, obstructive sleep apnea (OSA), and obesity presented to the emergency department for progressively worsening upper and lower extremity muscle weakness of two weeks' duration. He described having difficulty getting up from his chair, raising his arms, and being unable to walk. He reported having general muscle soreness and recent weight loss. He denied any trauma, overexertion in the heat, animal bites, illicit drug use, or recent illness. He denied any dysphagia, dysphonia, or signs of respiratory muscle weakness. His medications included losartan 50 mg daily, amlodipine 10 mg daily, atorvastatin 20 mg daily, metformin 500 mg daily, and dulaglutide 1.5 g/0.5 mL daily. He had been on atorvastatin for 1.5 years and was not initiated on any alternative statin therapy. His only recent change in medication was an increased dose of dulaglutide. 

On examination, the patient was normotensive with a blood pressure of 121/57 mmHg, temperature of 96.8°F, heart rate of 92 beats per minute, respiratory rate of 16 breaths per minute, and SpO2 100%. His physical exam showed weakness that was worse in the proximal lower extremities with no pain to palpation of the upper or lower extremities. Based on manual muscle testing grades, the lower extremity showed 3/5 strength for bilateral hip flexion, with 4+/5 strength globally of the bilateral upper extremity. All other aspects of the physical exam were unremarkable.

**Figure 1:**
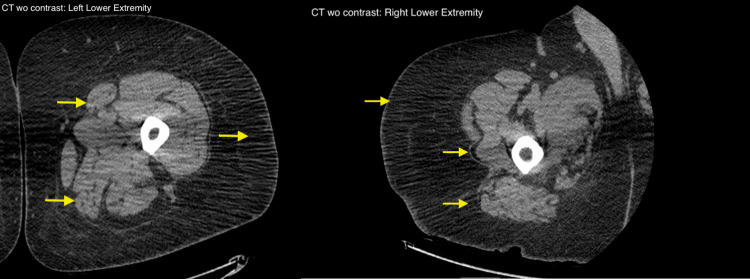
CT without contrast of bilateral lower extremities showing muscular atrophy and subcutaneous edema

His labs upon admission showed a complete blood count (CBC) with elevated RBCs at 6.12 M/uL, WBC at 8.4 K/uL, and hemoglobin 15.7 g/dL. Comprehensive metabolic panel (CMP) showed a sodium of 132 mmol/L, chloride of 94 mmol/L, glucose of 489 mg/dL, creatinine of 0.67 mg/dL, aspartate aminotransferase (AST) of 239 U/L, and alanine transaminase (ALT) of 384 U/L. Initial creatine kinase (CK) was 13,709 U/L. Urinalysis showed glucosuria, ketonuria, proteinuria, and microscopic hematuria. Initial troponin at 757 ng/L with two-hour troponin at 644 ng/L (Table 1). CT of bilateral lower extremities showed mild bilateral subcutaneous edema and mild bilateral muscular atrophy (Figure 1).

**Table 1:** Key laboratory assessments

Laboratory Assessments	Patient Values	Reference Ranges
White Blood Cell (WBC)	8.4	3.8-10 thousand/uL
Red Blood Cell (RBC)	6.12	4.20 -5.80 million/uL
Hemoglobin	15.7	13.2-17.1 g/dL
Sodium	132	135-146 mmol/L
Chloride	94	98-110 mmol/L
Glucose	489	65-99 mg/dL
Aspartate aminotransferase (AST)	239	10- 40 U/L
Alanine transaminase (ALT)	384	9-46 U/L
Creatine Kinase (CK)	13709	44-196 U/L
Initial Troponin T, 5th generation	757	≤ 15 ng/L
2-Hour Troponin T, 5th generation	644	≤ 15 ng/L

Given the elevated CK and transaminases along with clinical symptoms, the statin was held for a possible statin-induced myopathy. The patient was started on fluid resuscitation with an initial 1L bolus of Lactated Ringer's followed by initiation of 0.9% normal saline (NS) at 150 ml/hour. An abdominal ultrasound was ordered, and CK was trended. A cardiac workup was also completed to rule out acute coronary syndrome due to the elevated troponins, which included starting a heparin drip and obtaining an echocardiogram that showed a normal ejection fraction with no left ventricular hypertrophy. Initial EKG showed sinus tachycardia, left axis deviation, poor R-wave progression, and mildly diffuse ST elevation. 

**Figure 2:**
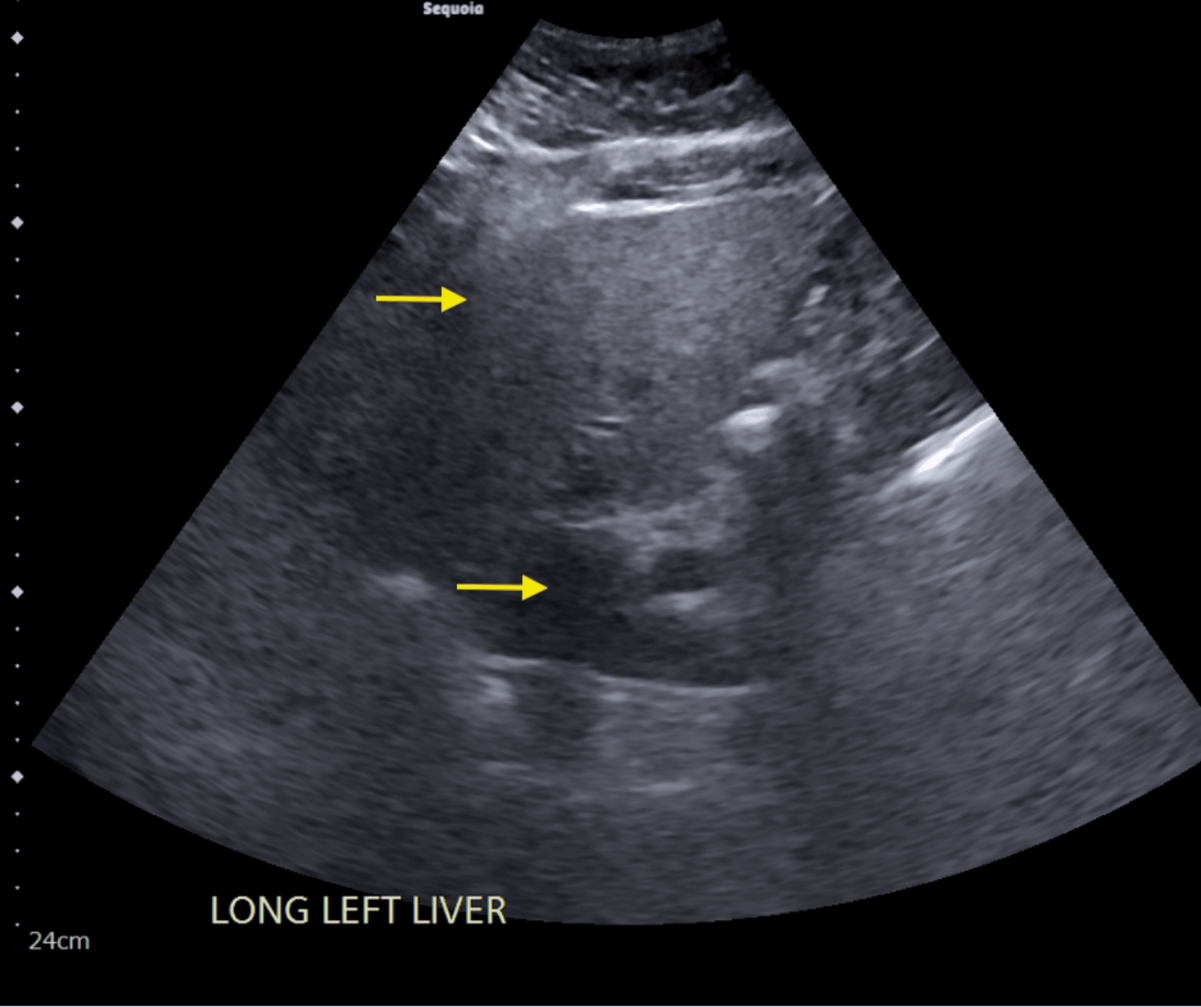
Ultrasound of liver showing mild hepatomegaly and hepatic steatosis

**Figure 3:**
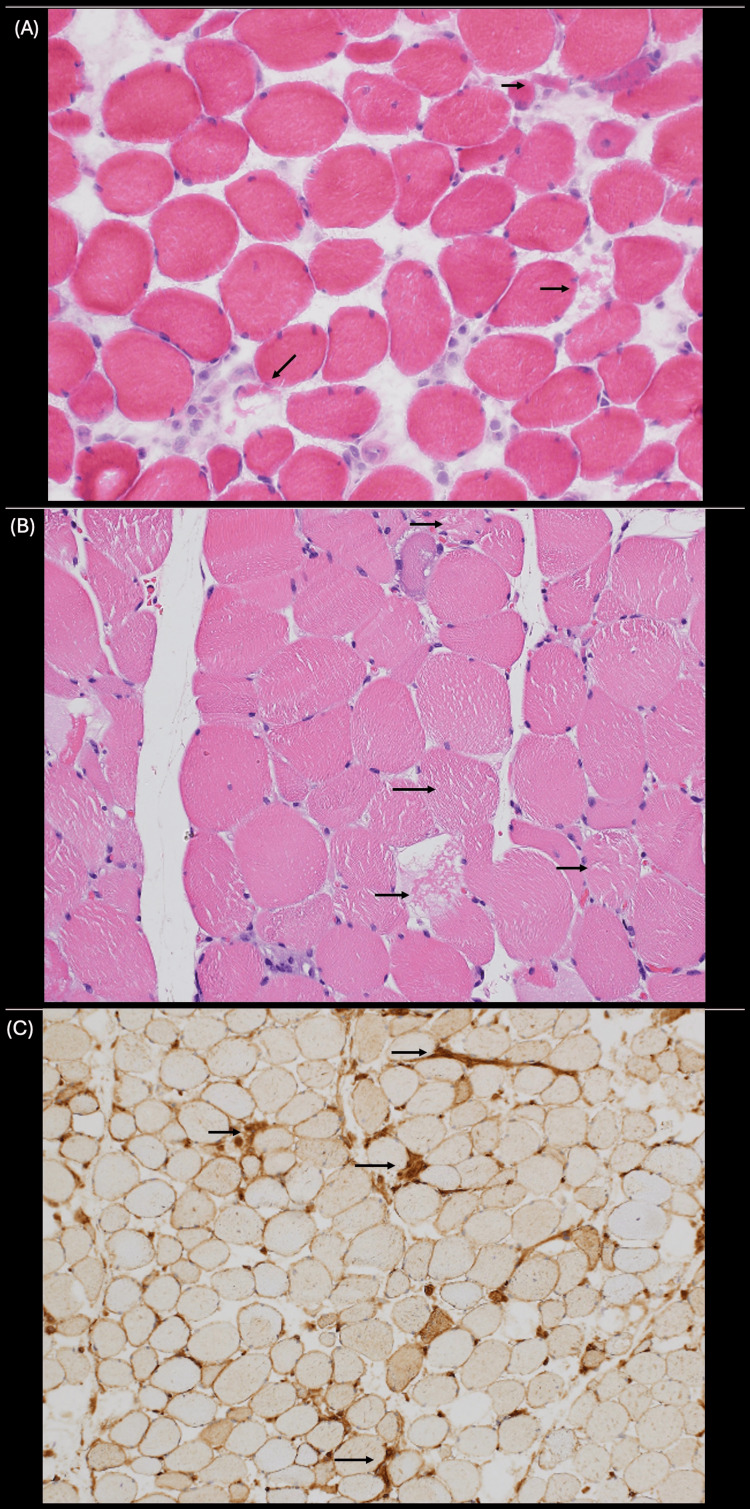
Histological analysis of muscle biopsy. (A) H&E-stained section, 400x original magnification, demonstrates scattered, occasional necrotic and regenerating myofibers were present without significant chronic lymphoid inflammation (arrows). (B) H&E-stained formalin-fixed paraffin block section, 400x original magnification, demonstrates scattered occasional necrotic and regenerating myofibers were present without significant chronic lymphoid inflammation (arrows). (C) MHC1 immunohistochemically stained section, 100x original magnification, demonstrates a diffuse mild to moderate increase in sarcolemmal staining of muscle fibers with accentuation of sarcolemmal and sarcoplasmic staining in regenerating and/or necrotic myofibers (arrows).

Despite proper fluid resuscitation and discontinuation of statin therapy, CK continued to remain elevated. The abdominal ultrasound showed mild hepatomegaly and hepatic steatosis but no acute findings to explain the elevated transaminases and CK (Figure 2). A muscle biopsy and myositis panel were ordered to investigate an autoimmune myopathy. The patient was started on prednisone, 60 mg daily. CK levels began to decline, and the patient reported improvement in symptoms. Muscle biopsy suggested immune-mediated necrotizing myopathy, and an extended myositis panel was HMGCR antibody positive (Figure 3).

The patient was discharged with follow-up with rheumatology, where steroids were continued. Given the continued elevation of transaminases, he was started on mycophenolate mofetil. To further control muscle inflammation, intravenous immunoglobulin (IVIG) was considered; however, the patient declined treatment with IVIG due to religious reasons. Therefore, he was started on rituximab as an alternative to IVIG.

## Discussion

In the cholesterol biosynthesis pathway, HMGCR is a glycoprotein that catalyzes the conversion of HMG-CoA to mevalonic acid [5]. These glycoproteins are the targets for statins, which are HMGCR inhibitors, to reduce cholesterol levels in patients with hyperlipidemia who are at high risk for cardiovascular events or to reduce future cardiovascular events in patients with established cardiovascular disease [5,6]. Although the exact pathophysiology is still unclear, particularly in the role of statins, anti-HMGCR antibodies, along with a possible association with class II HLA allele DRB1*11:01, result in an autoimmune response against skeletal muscles that causes scattered muscle necrosis and regeneration, leading to the clinical characteristics of muscle weakness seen in IMNM. The disease’s onset varies from a few weeks to more than 6 months, along with a long disease duration, especially if not treated early [7,8].

While the primary characteristic of anti-HMGCR IMNM is proximal muscle weakness, it is also important to note the impact it may also have on cardiac muscles. While this case presented with elevated troponin and an otherwise unremarkable cardiac workup, the troponin remained elevated months later, suggesting there is possible cardiac involvement related to the anti-HMGCR IMNM. However, no further cardiac workup was completed to determine whether the troponin elevation was related to the inflammatory myopathy within skeletal muscle or whether cardiac muscles were also involved. There have been a few case reports and studies that explored cardiac abnormalities in patients with IMNM. These studies have reported elevated troponin, EKG abnormalities, systolic dysfunction, diastolic dysfunction, and myocardial inflammation that developed in patients with anti-HMGCR IMNM and showed improvement with treatment of anti-HMGCR IMNM [9-11]. Further studies are needed to explore the prevalence of cardiac involvement in this population and whether this would develop any long-term cardiovascular issues for these patients. For the time being, it is important to emphasize the need to start cardiac workup in any patient who presents with anti-HMGCR IMNM.

Early recognition and proper treatment are also crucial for patients with anti-HMGCR IMNM. However, establishing a treatment regimen for anti-HMGCR IMNM can be difficult due to the lack of an established protocol. Despite the rarity of anti-HMGCR IMNM, the patient’s statin was stopped early in the admission, despite not having a definite diagnosis. Given the association of statins with anti-HMGCR IMNM, stopping statins should be considered for any patient presenting with unexplained muscle weakness and elevated CK. After cessation of the possible offending agent, there is no clear literature that provides a definite treatment regimen regarding immunosuppressive treatment. However, the 224th ENMC International Workshop provided clinicians with a possible approach to treating anti-HMGCR IMNM [12].

Nearly all cases should start treatment with a high-dose oral corticosteroid [12,13]. Ideally, if the patient responds well to the steroid treatment, the treatment can be tapered as symptoms are resolved, as the patient is started on a disease-modifying antirheumatic drug (DMARD) such as azathioprine, methotrexate, mycophenolate mofetil, or rituximab [3,12]. However, in this case, despite improvement of CK levels with initiation of corticosteroid, it was not tapered as his muscle weakness did not significantly improve. Methotrexate is usually used as first-line treatment unless there are any adverse side effects or contraindications, such as the elevated LFT and hepatic steatosis seen in our patient. Azathioprine or mycophenolate mofetil can be used as a second-line treatment for anti-HMGCR IMNM for those with contraindications to first-line treatments [12].

In addition to DMARDs, experts also suggest that IVIG should be used in the treatment of anti-HMGCR IMNM. However, when IVIG should be initiated and the length of treatment is still uncertain. There are some recommendations that IVIG should be first line, while others suggest it at the same time as methotrexate initiation [12]. Additionally, rituximab has also been suggested as a third-line treatment or for refractory anti-HMGCR IMNM, although it has been more commonly used in anti-signal recognition particle IMNM [12,13]. Double or triple agent treatment may be needed for patients with anti-HMGCR IMNM who present with a severe disease or have a longer disease duration. Without controlled trials to compare the efficacy of these treatments, it is difficult to provide a clear guideline for the treatment of anti-HMGCR IMNM. However, this case presentation provides an example of how the regimen of steroids, mycophenolate mofetil, and rituximab can be used to treat anti-HMGCR IMNM. 

## Conclusions

Anti-HMGCR IMNM is a rare subtype of autoimmune myopathies that presents with proximal muscle weakness and elevated CK. The growing use of statin today emphasizes the importance of the recognition of this disease by providers of all specialities so that early diagnosis can be made and treatment is not delayed. However, lack of clear evidence-based guidelines for the treatment of this disease can make it difficult to have full recovery of symptoms for some patients. It is also unclear on the effect that anti-HMGCR IMNM has on the heart, especially its long-term effects. As anti-HMGCR IMNM becomes more recognized, further research will need to be done to develop treatment regimens as well as the long-term effects anti-HMGCR has on the body. 
